# Autophagy-Related Long Non-coding RNA Is a Prognostic Indicator for Bladder Cancer

**DOI:** 10.3389/fonc.2021.647236

**Published:** 2021-04-01

**Authors:** Jiaming Wan, Cheng Guo, Hongpeng Fang, Zhongye Xu, Yongwei Hu, Yun Luo

**Affiliations:** ^1^Department of Urology, The Third Affiliated Hospital of Sun Yat-sen University, Guangzhou, China; ^2^Department of Otorhinolaryngology Head and Neck Surgery, The Third Affiliated Hospital of Sun Yat-sen University, Guangzhou, China

**Keywords:** autophagy, TCGA, bladder cancer, prognostic indicator, lncRNA

## Abstract

Bladder cancer (BC) is one of the most common malignant urinary system tumors, and its prognosis is poor. In recent years, autophagy has been closely linked to the development of BC. Therefore, we investigated the potential prognostic role of autophagy-related long non-coding RNA (lncRNA) in patients with BC. We obtained the lncRNA information and autophagy genes, respectively, from The Cancer Genome Atlas (TCGA) data set and the human autophagy database (HADb) and performed a co-expression analysis to identify autophagy gene-associated lncRNAs. Then, we divided the data into training group and testing group. In the training group, 15 autophagy-related lncRNAs were found to have a prognostic value (AC026369.3, USP30-as1, AC007991.2, AC104785.1, AC010503.4, AC037198.1, AC010331.1, AF131215.6, AC084357.2, THUMPD3-AS1, U62317.4, MAN1B1-DTt, AC024060.1, AL662844.4, and AC005229.4). The patients were divided into low-risk group and high-risk group based on the prognostic lncRNAs. The overall survival (OS) time for the high-risk group was shorter than that for the low-risk group [risk ratio (hazard ratio, HR) = 1.08, 95% CI: 1.06–1.10; *p* < 0.0001]. Using our model, the defined risk value can predict the prognosis of a patient. Next, the model was assessed in the TCGA testing group to further validate these results. A total of 203 patients with BC were recruited to verify the lncRNA characteristics. We divided these patients into high-risk group and low-risk group. The results of testing data set show that the survival time of high-risk patients is shorter than that of low-risk patients. In the training group, the area under the curve (AUC) was more than 0.7, indicating a high level of accuracy. The AUC for a risk model was greater than that for each clinical feature alone, indicating that the risk value of a model was the best indicator for predicting the prognosis. Further training data analysis showed that the gene set was significantly enriched in cancer-related pathways, including actin cytoskeleton regulation and gap junctions. In conclusion, our 15 autophagy-related lncRNAs have a prognostic potential for BC, and may play key roles in the biology of BC.

## Introduction

Global Cancer Observatory (GCO) data (https://gco.iarc.fr/) (1) show that ~550,000 people were diagnosed with bladder cancer (BC) in 2018, making BC the 10th common and 13th most fatal tumor worldwide. Of all patients with BC, ~77% are male and BC is the sixth most common cancer in males worldwide. Furthermore, 53% of the patients with BC are more than 70 years old and the mortality is as high as 47%. The diagnosis of BC is mainly dependent on the findings of specimen biopsy, and the treatments for BC have generally included radical/partial cystectomy, neoadjuvant chemotherapy, adjuvant chemotherapy/radiation, and checkpoint inhibitor-based targeted therapies (2–6). BC is considered to be one of the tumors possessing the greatest economic burden on its treatment because of the difficulty of an accurate diagnosis and a precise treatment, inevitable frequent surveillance strategies, and the high recurrence risk (7, 8). Therefore, timely diagnosis and clinical staging of BC for enabling reasonable and effective treatment measures become an urgent problem.

Autophagy is a process of elemental catabolism responsible for intracellular degradation. In the process of autophagy, excessive, aggregated, or damaged proteins and organelles are transferred to lysosomes for the degradation and reuse, thereby producing macromolecular molecules and energy (9, 10). Ishaq and his colleagues (11) provided a good overview of the role of autophagy in tumors, describing that autophagy can exert anticancer and pro-cancer dual effects by affecting the web of inter-organelle membrane contact sites, cancer stem cells, cancer metabolism, and tumor immunity (11). Moreover, autophagy is also involved in gene repair and supports the synthesis of DNA in response to oncogenes (12, 13). Few studies show that microRNA (miRNA) 516a exerts carcinogenic effects in BC by attenuating beclin-1 (BECN1)-dependent autophagy (14), the miR-139-5p/Bmi-1 axis is closely related to AMP activated protein kinase (AMPK) and mammalian target of rapamycin (mTOR) pathway-dependent autophagy in human BC cells (15), and autophagy-related gene 7 (ATG7) promotes the invasion of BC *via* an autophagy-mediated increase in the rho GDP dissociation inhibitor beta (*ARHGDIB*) messenger RNA (mRNA) stability (16). Although the role of autophagy and targeted therapy strategies based on this process have gradually become a research hotspot in BC in recent years, the involvement of specific mechanisms and checkpoints remains controversial.

Since the development of high-throughput genomics, long non-coding RNA (lncRNA) has been validated as a non-protein coding RNA comprising different types of RNA polymerase II (Pol II)-transcribed molecules longer than 200 nucleotides (17, 18). lncRNA participates in the occurrence and development of cancer by regulating biological processes including cell growth, cell cycle, cell metastasis, cell death, angiogenesis, metabolic disorders, drug resistance, immune escape, DNA damage, and cell stemness (19, 20). Many reports have corroborated the association between lncRNA and autophagy in cancer. BLACAT1, MALAT1, XIST, SNHGs, HULC, CASC2, GAS5, H19, and HOTAIR all are lncRNAs that mediate chemoresistance in the tumor by modulating autophagy (19). The expression of lncRNA maternally expressed 3 (MEG3) leads to a reduced autophagy activation, which reduces the proliferation of BC cells (21). lncRNA urothelial cancer associated 1 (UCA1) acts as an oncogene and promotes malignant progression of BC through the UCA1-miR-582-5p-ATG7 autophagy signaling pathway (22). Therefore, autophagy-related lncRNAs may be used as prognostic factors in patients with BC and also as potential therapeutic targets. Here, we strived to establish a prognostic system for autophagy-related lncRNA in BC and to promote the targeted therapy of BC.

## Materials and Methods

### The Extraction of Information About Patients With BC

The Cancer Genome Atlas (TCGA; https://cancergenome.nih.gov/) microarray was used as a training group to establish an autophagy-related lncRNA signature in patients with BC. TCGA is a cancer and tumor gene mapping program launched in the USA in 2006 (23). TCGA includes thousands of samples that have been sequenced by the whole transcriptome sequencing, DNA methylation microarray detection, miRNA, mRNA, and other sequencing. The training data set includes TCGA RNA sequencing (RNA-seq) [fragments per kilobase of transcript per million (FPKM)] from 411 patients with bladder urothelial carcinoma (BLCA), a type of BC, and related clinical data. Transcriptome data and clinical information were downloaded from the data set website.

### Screening for lncRNA Co-expressed With Autophagy-Related Genes

Autophagy-related genes were extracted from the human autophagy database (HADb, http://autophagy.lu/clustering/index.html). The Limma R package was used to extract the autophagy-related gene expression information from the TCGA-BLCA RNA sequencing data. To identify the lncRNAs-targeting autophagy-related genes, we analyzed the Pearson correlation between the lncRNA and autophagy-related gene expression levels using the screening criteria: (| R2 | > 0.3 and *p* < 0.001). Finally, 1435 lncRNAs co-expressed with autophagy-related genes were identified in a TCGA-BLCA data set.

### Establishment of a Risk Score Model and Building a Risk Curve

We use the survival R package to construct the lncRNA signature. The BLCA sample criteria for identifying and verifying lncRNA characteristics were: (1) complete lncRNA expression value; and (2) complete clinical characteristics (the survival time and survival status). A total of 406 BLCA samples were included for further construction of lncRNA characteristics. We randomly divided the 406 BLCA samples into the training group (*n* = 203) and testing group (*n* = 203). The training cohort was used to develop the lncRNA features. The experimental cohort was used to verify the lncRNA characteristics.

Secondly, 406 lncRNAs were selected from a training cohort for univariate Cox regression analysis to identify prognosis-related lncRNAs (*p* < 0.005). Univariate Cox regression analysis identified 45 prognostic lncRNAs. We used the Akaike information criterion (AIC) method to select the optimal model from 45 prognostic lncRNAs. Finally, we selected 15 lncRNAs with the lowest AIC values to build a prediction model.

Multivariate Cox regression analysis was performed on 15 prognostic lncRNAs to determine their prognostic characteristics and calculate the correlation coefficient (24, 25). The risk score for the prognostic lncRNA characteristics of each patient was calculated by using the following formula:

$$\begin{array}{l} \text{Risk score }=\text{expression lncRNA}1\;\times \text{ coefficient lncRNA}1\\ \;+\text{ expression lncRNA}2\;\times \text{ coefficient lncRNA}2\;+\;\ldots \\ \;+\text{ the expression lncRNAn }\times \text{ coefficient lncRNAn}. \end{array}$$

The risk score of prognostic lncRNAs was calculated by using a linear combination of lncRNA expression levels weighted by a regression coefficient (β). β was calculated by a log conversion of the hazard ratio (HR) obtained from multivariate Cox regression analysis (26). Low-risk group and high-risk group were determined by using the median-risk score.

### Risk Model Prediction Ability in Training Group and Testing Group

Survival and phopmap packages were used to evaluate the predictive value of the risk model in a training cohort (*n* = 203). The training cohort was divided into low-risk group and high-risk group based on the median risk score. The Kaplan–Meier (KM) survival curve was drawn to compare the difference in the overall survival (OS) between the two groups and a risk curve was drawn. *p* < 0.05 was considered as statistically significant.

Subsequently, the training group was used to sort the complete data (*n* = 172) for each clinical feature. These clinical features include age, gender, union for international cancer control (UICC) stage, histologic grade, and pathological TNM (pTNM) stage. Univariate and multivariate Cox regression analyses were used to evaluate the predictive power of the risk model. Then, survivroc was used to draw the time-dependent receiver operating characteristic (ROC) curve and area under the curve (AUC) values to evaluate the predictive value of the comparative risk model and the assessed clinical features. *p* < 0.05 was considered as statistically significant. We then conducted a correlation analysis between the model risk score and the clinical characteristics of the training group. Each clinical feature was divided into two categories (e.g., dividing age into > 65 and ≤ 65, and dividing the grade into high and low). SPSS 25 software was used for statistical analyses.

The same method was used in the testing group to verify the stability and reliability of the risk model. The testing group (*n* = 203) was divided into low-risk group and high-risk group based on the median risk. The KM survival and risk curves were drawn. The testing group data with complete clinical data was selected (*n* = 172), and the time-dependent ROC curve and AUC value were determined to evaluate the predictive value of the comparative risk model and these clinical features.

### Gene Set Enrichment Analysis

Gene set enrichment analysis (GSEA) was used to determine the gene set functions. The aim was to use a predefined set of genes, sort the genes according to the differential expression levels in the two sample groups, and check whether the gene set was enriched at the top or bottom of this sorting table.

### Statistical Analysis

Cytoscape software (version 3.7.2) was used to construct an autophagy-related lncRNA co-expression network. R language (version x64 3.6.3, survival library) was used to estimate the survival rate, generate a survival curve, and perform the Cox regression analysis. We used the R language (pheatmap library) to generate the risk value, survival time, and risk heat maps. We used the R language (survival ROC library) to draw a multi-index ROC curve. GSEA (version 4.0.3) was used to distinguish between the two sets of functional annotations. SPSS 25 was used to analyze the correlation between the risk model and clinical features. Statistical significance was set at the threshold of *p* < 0.05 for both tails.

## Results

### Autophagy-Related Gene lncRNAs Co-expression Network Construction

**Figure 1:**
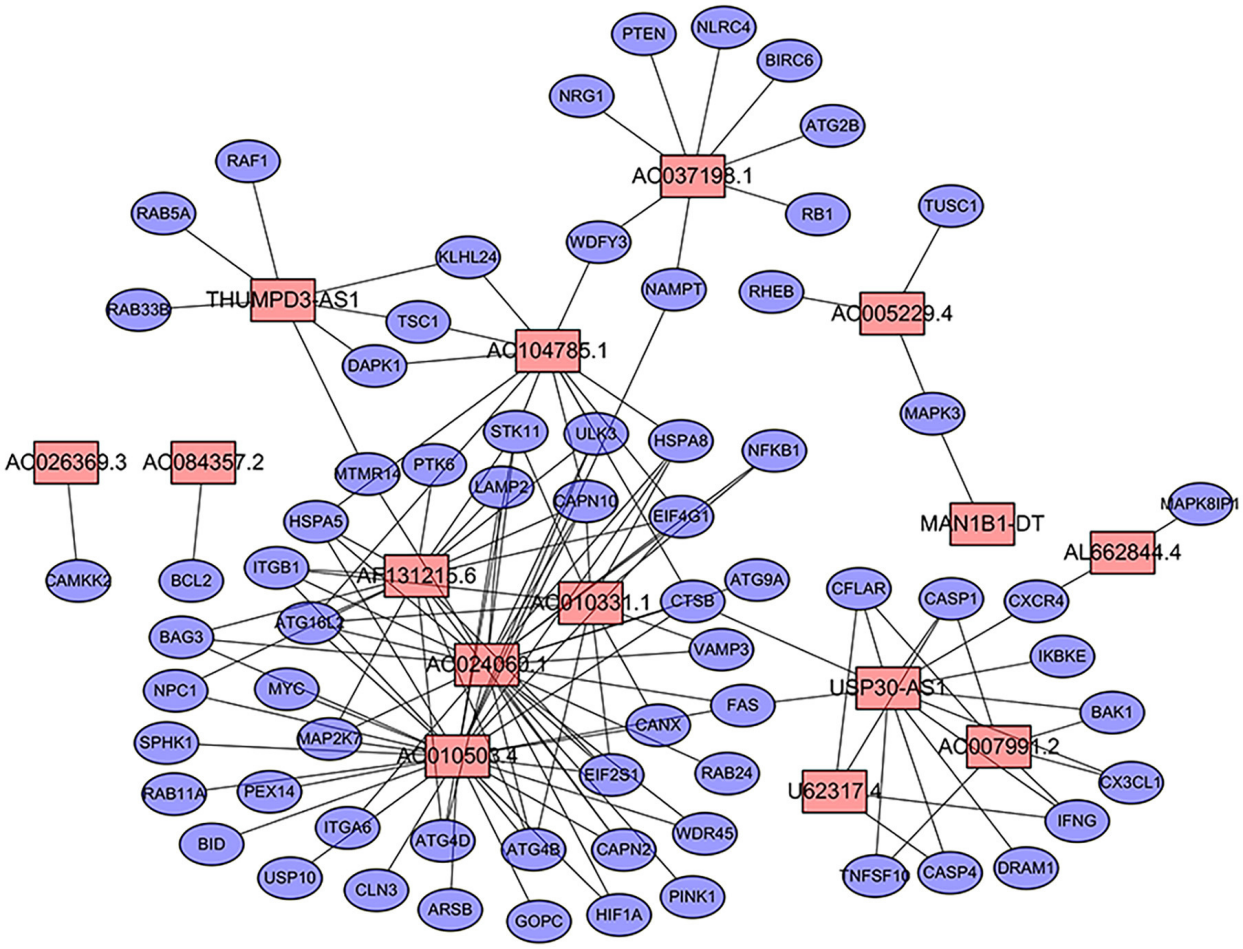
Prognostic network of long non-coding RNAs (lncRNAs) and co-expressed autophagy genes in bladder cancer (BC). In the center, the red node represents lncRNA, and the blue represents autophagy genes. The co-expression network was constructed by using CYTOSCAPE 3.7.2 software.

We extracted the lncRNA data from the TCGA database transcriptome data set. A total of 232 autophagy-related genes were extracted from the HADb (http://autophagy.lu/clustering/index.html). We constructed an autophagy-related lncRNA co-expression network and performed a co-expression analysis to identify autophagy-related lncRNAs. The screening criteria were (| R2 | > 0.3 and *p* < 0.001). A co-expression network diagram with autophagy genes was drawn by using the 15 prognostic-related lncRNAs identified by multivariate Cox analysis (Figure 1).

### Selection of 15 Autophagy-Related Prognostic lncRNAs for Patients With BC

First, we used univariate Cox regression analysis based on the identified autophagy-related lncRNAs to initially screen the prognostic genes. We used a value of *p* = 0.005 as a cutoff value, and the lncRNAs that met this criterion were used to judge prognostic indicators. Our training set included 203 patients with BC from the TCGA data set. A total of 45 lncRNAs had a prognostic value for patients with BC (*p* < 0.01) (Table 1). Then, we used the AIC method to select the best model from the 45 prognostic lncRNAs. About 15 autophagy-related lncRNAs were found to be suitable for model building. Of these lncRNAs, 5 were unfavorable prognostic factors (AC037198.1, AC084357.2, MAN1B1-DT, AC024060.1, and AC005229.4) and 10 were favorable prognostic factors (AC026369.3, USP30-as1, AC007991.2, AC104785.1, AC010503.4, AC010331.1, AF131215.6, THUMPD3-AS1, U62317.4, and AL662844.4) for BC (Table 2). These 15 autophagy-related lncRNAs were used as a prognostic model.

**Table 1:** Autophagy-related long non-coding RNAs (lncRNAs) (*n* = 45) significantly associated with the prognosis of bladder cancer (BC).

**ID**	**HR**	**95% CI**	***P*-value**
AC026369.3	0.285	0.120–0.675	0.004
USP30-AS1	0.601	0.447–0.806	<0.001
AC025165.4	0.360	0.178–0.729	0.005
AC007991.2	0.609	0.436–0.851	0.004
AC104785.1	0.389	0.202–0.747	0.005
AC112721.2	1.376	1.148–1.649	<0.001
AC010503.4	0.754	0.639–0.889	<0.001
AC037198.1	1.475	1.141–1.907	0.003
LINC00942	1.278	1.102–1.481	0.001
AC116914.2	0.521	0.337–0.807	0.004
TNFRSF14-AS1	0.516	0.336–0.791	0.002
MAFG-DT	1.464	1.128–1.900	0.004
AC010331.1	0.481	0.297–0.776	0.003
MANCR	1.524	1.199–1.938	<0.001
AF131215.6	0.582	0.410–0.824	0.002
LINC00324	0.529	0.348–0.803	0.003
SPINT1-AS1	0.738	0.599–0.910	0.005
AC005840.4	0.438	0.274–0.701	<0.001
DBH-AS1	0.427	0.246–0.742	0.003
AC112721.1	1.362	1.133–1.638	0.001
AC016957.2	0.394	0.209–0.745	0.004
AC104825.1	0.663	0.499–0.880	0.004
AC022150.2	0.514	0.359–0.736	<0.001
LINC01871	0.707	0.576–0.867	<0.001
ASB16-AS1	0.461	0.284–0.749	0.002
MIR200CHG	0.777	0.673–0.897	<0.001
AC008610.1	0.555	0.382–0.807	0.002
CARD8-AS1	0.406	0.229–0.719	0.002
AC084357.2	2.242	1.397–3.597	<0.001
LINC01356	1.430	1.138–1.797	0.002
AL033397.1	1.402	1.148–1.714	<0.001
AC009283.1	0.660	0.496–0.878	0.004
ATP1B3-AS1	1.786	1.215–2.626	0.003
AC005291.2	1.509	1.137–2.003	0.004
THUMPD3-AS1	0.546	0.366–0.815	0.003
AL450384.2	0.601	0.441–0.818	0.001
LINC02446	0.615	0.453–0.834	0.002
AC087741.1	0.547	0.365–0.821	0.004
IPO5P1	0.696	0.551–0.880	0.002
U62317.4	0.521	0.340–0.798	0.003
MAN1B1-DT	1.865	1.225–2.839	0.004
AC024060.1	0.628	0.458–0.861	0.004
AL662844.4	0.324	0.152–0.694	0.004
AC011374.2	0.482	0.296–0.784	0.003
AC005229.4	1.948	1.249–3.038	0.003

*HR, hazard ratio; CI, confidence interval*.

**Table 2:** Multivariate cox analysis revealed 15 autophagy lncRNAs that were significantly associated with BC prognosis.

**ID**	**COEF**	**HR**	**95% CI**
AC026369.3	−0.886	0.412	0.182–0.937
USP30-AS1	−0.329	0.720	0.469–1.105
AC007991.2	−0.488	0.614	0.398–0.947
AC104785.1	−0.739	0.478	0.205–1.115
AC010503.4	−0.308	0.735	0.570–0.946
AC037198.1	0.776	2.173	1.515–3.116
AC010331.1	−0.529	0.589	0.325–1.067
AF131215.6	−0.657	0.518	0.328–0.818
AC084357.2	0.978	2.659	1.686–4.192
THUMPD3-AS1	−0.715	0.489	0.247–0.967
U62317.4	−0.465	0.628	0.341–1.158
MAN1B1-DT	1.185	3.269	1.918–5.573
AC024060.1	0.796	2.216	1.240–3.958
AL662844.4	−2.014	0.133	0.042–0.427
AC005229.4	0.800	2.225	1.254–3.947

*COEF, coefficient; HR, hazard ratio; CI, confidence interval*.

### The Effect of Autophagy-Related lncRNA on BC Prognosis

**Figure 2:**
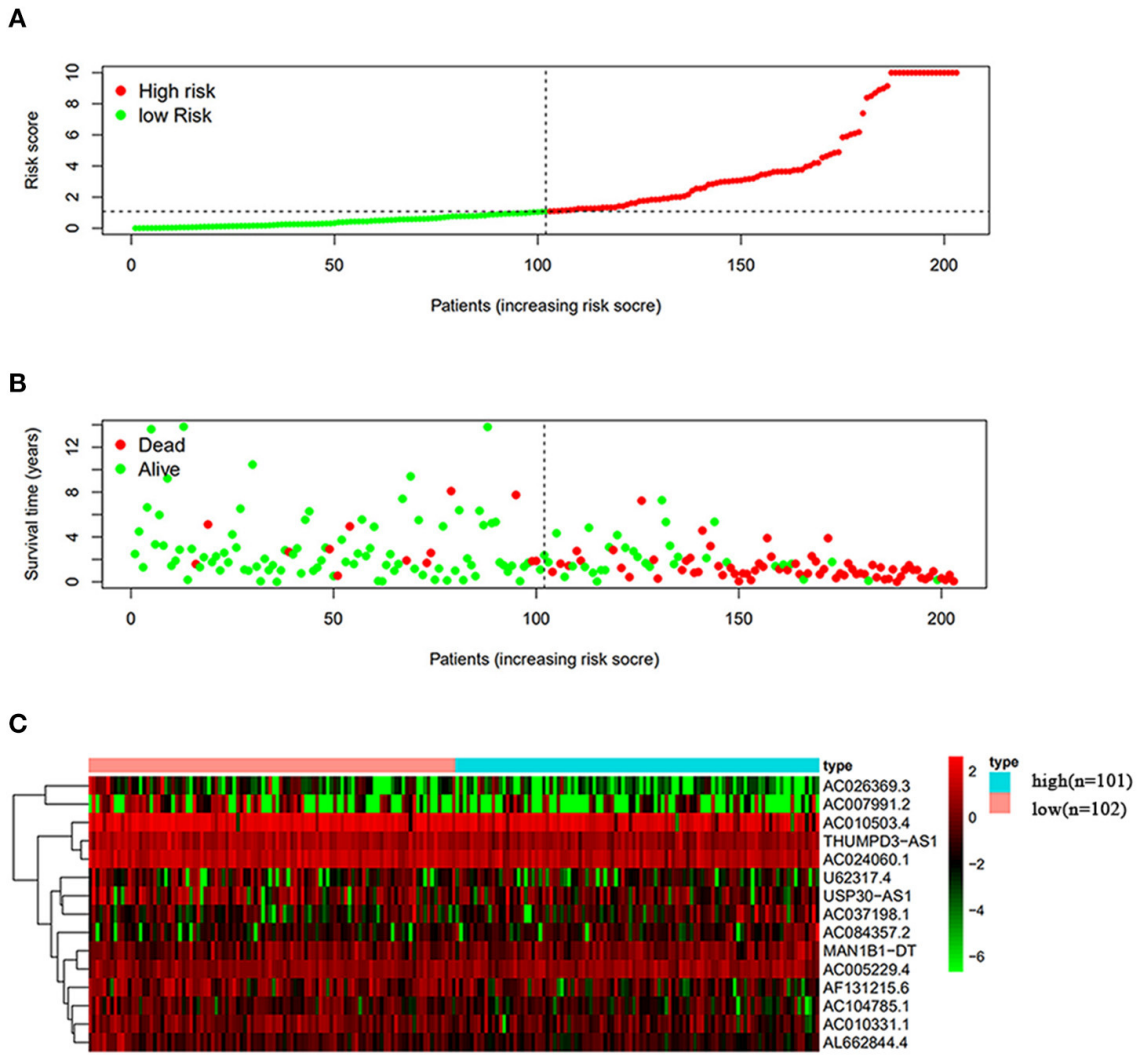
Analysis of autophagy-related lncRNA risk scores of patients with BC in The Cancer Genome Atlas (TCGA) training group. **(A)** Patients with BC were divided into low-risk group (*n* = 102) and high-risk group (*n* = 101) based on the median-risk score. **(B)** Survival status and duration of the survival in patients with BC. **(C)** Heat map of the expression of 15 key lncRNAs in BC. The color from green to red shows the expression trend from low level to high level.

**Figure 3:**
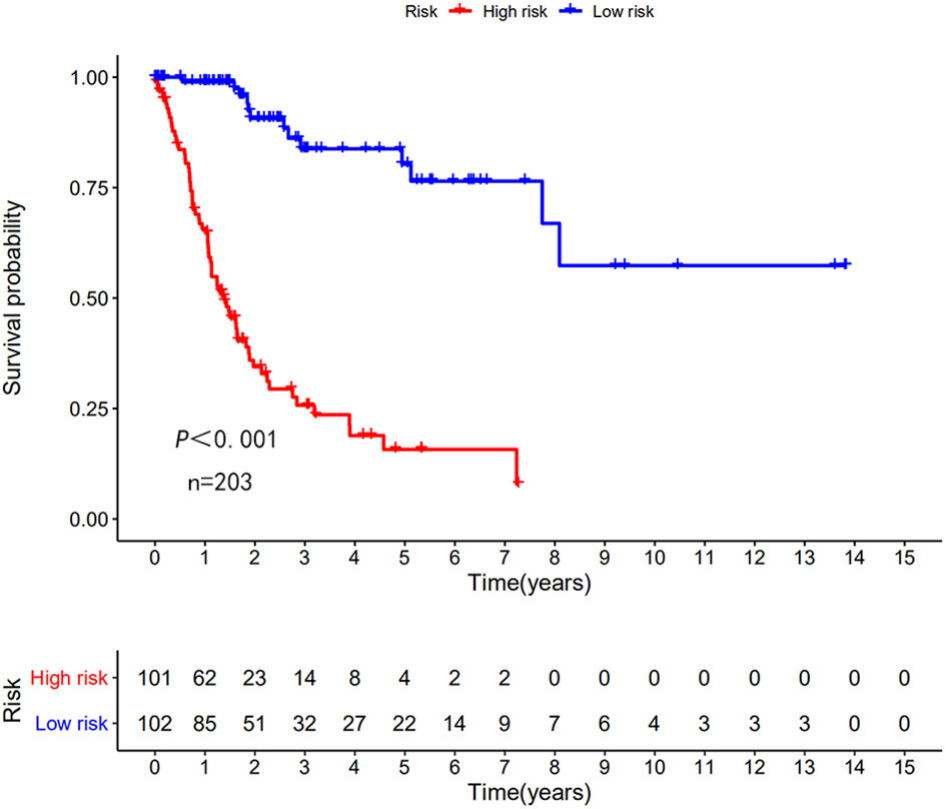
Kaplan–Meier (KM) survival curve of the autophagy-related lncRNA BC risk score in TCGA training group. In the TCGA data, the 5-year survival rate of high-risk patients is lower than that of low-risk patients.

**Figure 4:**
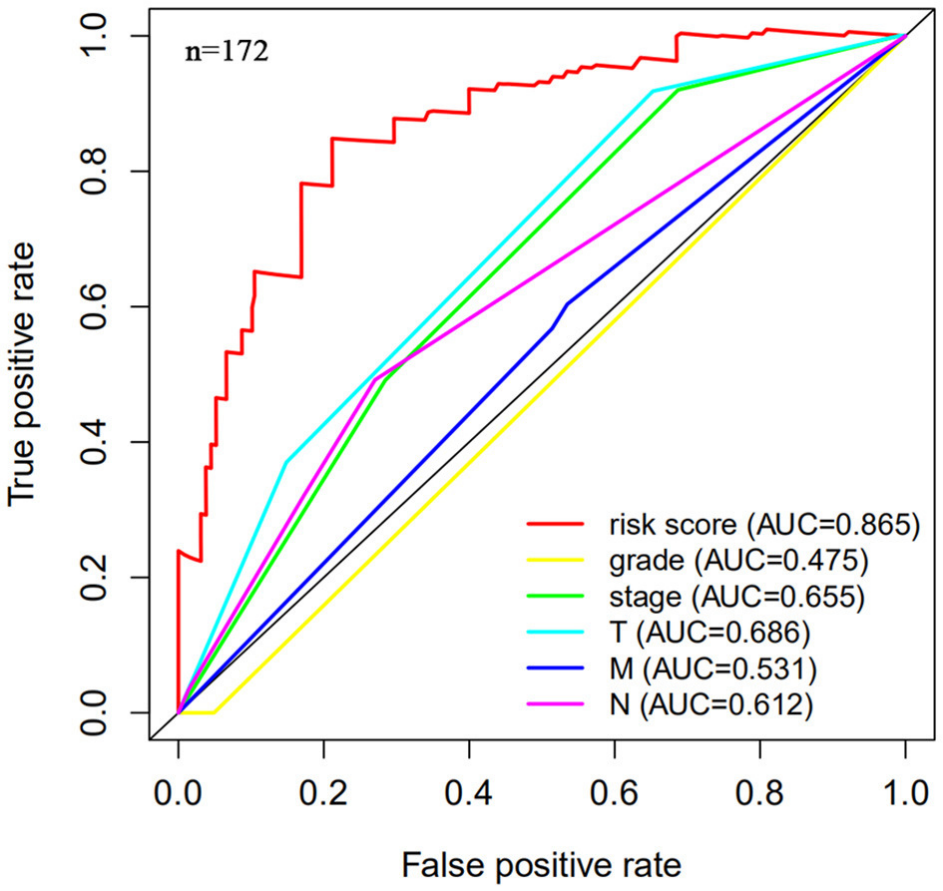
Multi-factor receiver operating characteristic (ROC) curve. The area under the curve (AUC) value of the model established in the training group is significantly more than 0.7, and is greater than the predicted value of clinical data.

The risk scoring method was used to assess the prognosis of BC in the training group (*n* = 203). We divided patients with BC into low-risk group and high-risk group based on the median risk score (Figure 2). The risk score can significantly predict the OS in patients with BC, with OS being longer in the low-risk group than in the high-risk group. Additionally, a KM survival curve for BC autophagy-related lncRNA risk scores was plotted (Figure 3). The KM survival curve showed that the 5-year survival rate was greater in the low-risk group than that in the high-risk group (log rank *p* < 0.01). We screened the training group data with complete clinical characteristics for the next analysis (*n* = 172) and used multivariate Cox regression analysis to explore if the risk score is an independent predictor of prognosis in patients with BC. The HR was 1.080, which indicates that the risk score model can significantly help predict the survival in patients with BC, and eliminates the effects of other factors (such as age, gender, and grade; Table 3). Next, the ROC curve of clinical data and risk score was drawn. The AUC of the ROC curve of the risk score was 0.865, which is > 0.7 and indicates that the model has good accuracy. The AUC of the model is greater than that of any other clinical characteristics, which indicate that the model is superior to other clinical traits in prognostic prediction (Figure 4).

**Table 3:** Multivariate Cox regression analysis of characteristics and risk score in BC.

**ID**	**B**	**SE**	***z***	**HR**	**95% CI**	***P*-value**
Age	0.040	0.014	2.906	1.040	1.013–1.069	0.004
Gender	−0.176	0.275	−0.637	0.839	0.489–1.440	0.524
Grade	1.910	1.099	1.738	6.754	0.784–58.181	0.082
Stage	0.268	0.323	0.830	1.307	0.694–2.459	0.407
T	0.462	0.239	1.935	1.588	0.994–2.536	0.053
M	0.226	0.139	1.628	1.253	0.955–1.645	0.103
N	0.138	0.215	0.640	1.148	0.753–1.749	0.522
Risk score	0.076	0.010	7.679	1.079	1.058–1.100	<0.001

*B, regression coefficient; SE, standard error; HR, hazard ratio; CI, confidence interval; stage, union for international cancer control (UICC) stage; T, pT stage; M, pM stage; N, pN stage*.

### Clinical Correlation Between Autophagy-Related lncRNAs and Patients With BC

**Figure 5:**
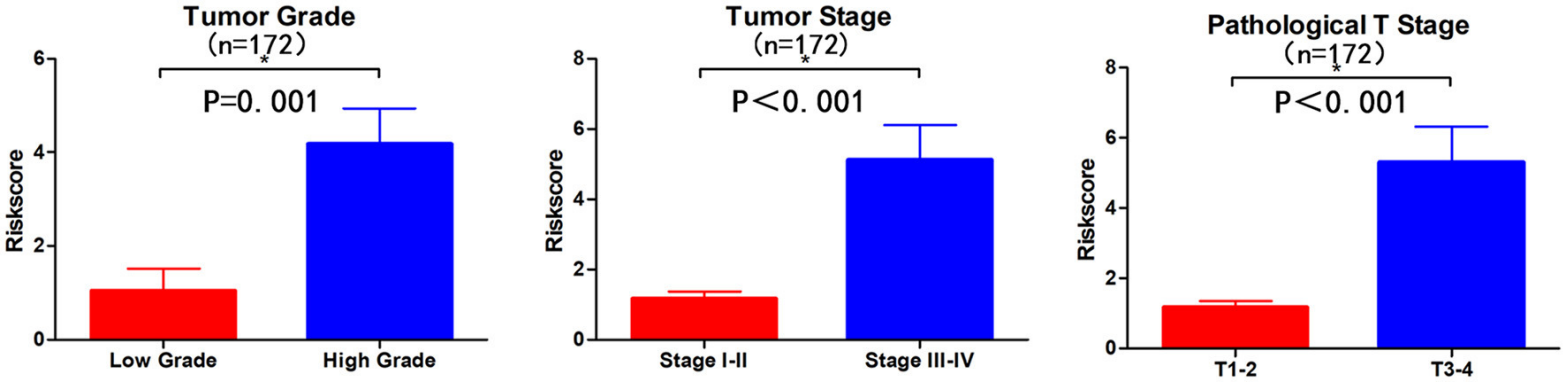
Visualization of the clinical correlation between autophagy-related lncRNA model and patients with BC. The risk score is closely related to grade, union for international cancer control (UICC) stage, and pathological T stage (*p < 0.05).

Subsequently, we determined the clinical value of the 15 autophagy-related lncRNAs in terms of age, gender, grade, and pTNM stage (Table 4). The risk score is closely related to grade, UICC stage, and pT stage (Figure 5), but is not strongly related to age, gender, pN stage, and pM stage. Additionally, the risk score tends to increase in higher grades, which indicates that the lncRNA signature may be related to BC progression.

**Table 4:** lncRNA clinical correlation analysis.

**Clinical**	**Group**	***N***	**Mean**	**SD**	***t***	***P*-value**
Age	>65	106	4.528	9.902	0.855	0.394
	≤65	66	3.287	8.838		
Gender	Female	54	5.305	12.264	1.003	0.319
	Male	118	3.478	7.921		
Grade	High	165	4.179	9.678	3.547	0.001
	Low	7	1.053	1.209		
Stage	I-II	47	1.17	1.374	−3.974	<0.001
	III-IV	125	5.135	10.929		
T	T1-2	52	1.163	1.323	−4.012	<0.001
	T3-4	120	5.303	11.123		
M	M0	78	4.203	11.032	0.185	0.853
	M1	94	3.926	8.073		
N	N0-1	138	3.663	8.909	−0.921	0.362
	N2-3	34	5.627	11.620		

*Clinical, clinical characteristics; Mean, arithmetic mean; SD, standard deviation; t, t-value; stage, UICC stage; T, pT stage; M, pM stage; N, pN stage*.

### Validation in the TCGA Data Set

**Figure 6:**
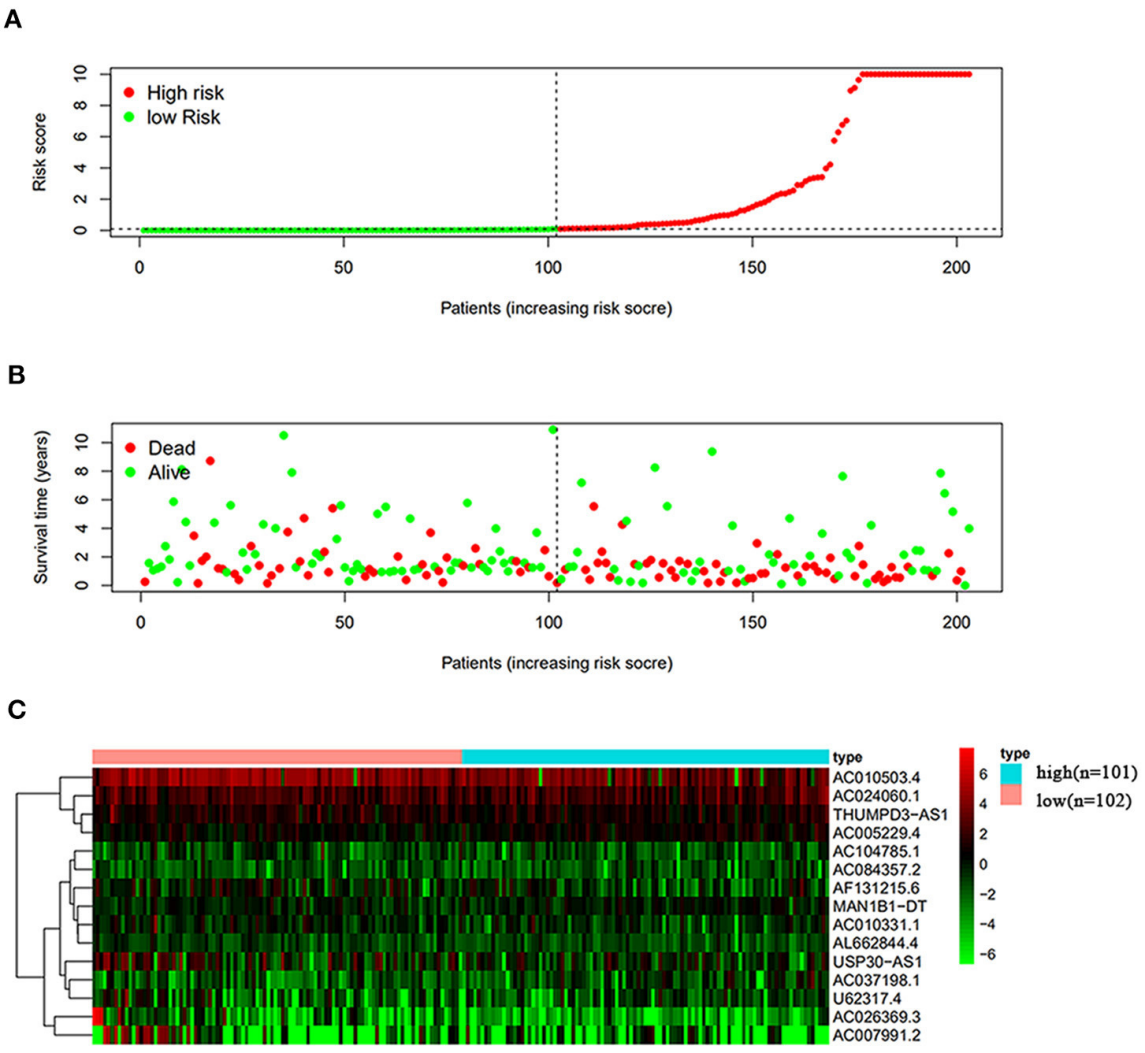
Analysis of autophagy-related lncRNA risk scores of patients with BC in TCGA testing group. **(A)** Autophagy-related lncRNA low-risk group (*n* = 102) and high-risk group (*n* = 101) in patients with BC. **(B)** Survival status and duration of BC cases. **(C)** Heat map of the expression of 15 key lncRNAs in BC. The color from green to red shows the expression trend from low level to high level.

**Figure 7:**
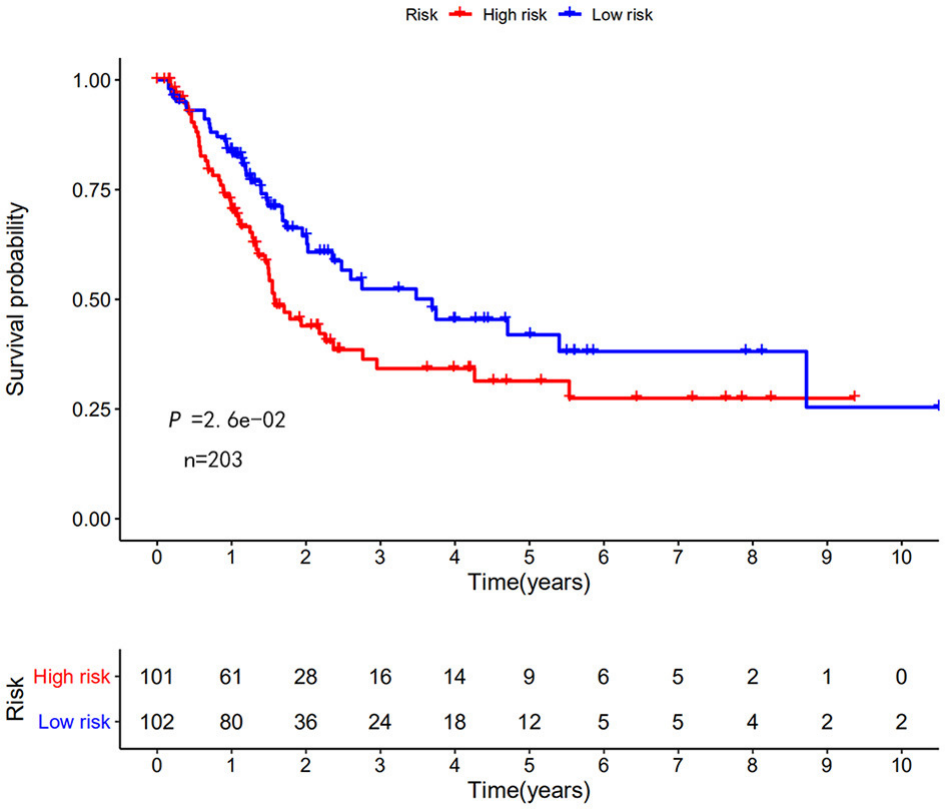
KM survival curve of the autophagy-related lncRNA risk score for BC in TCGA testing group. TCGA data shows that the 5-year survival rate of high-risk patients is lower than that of low-risk patients (log rank *p* < 0.01).

**Figure 8:**
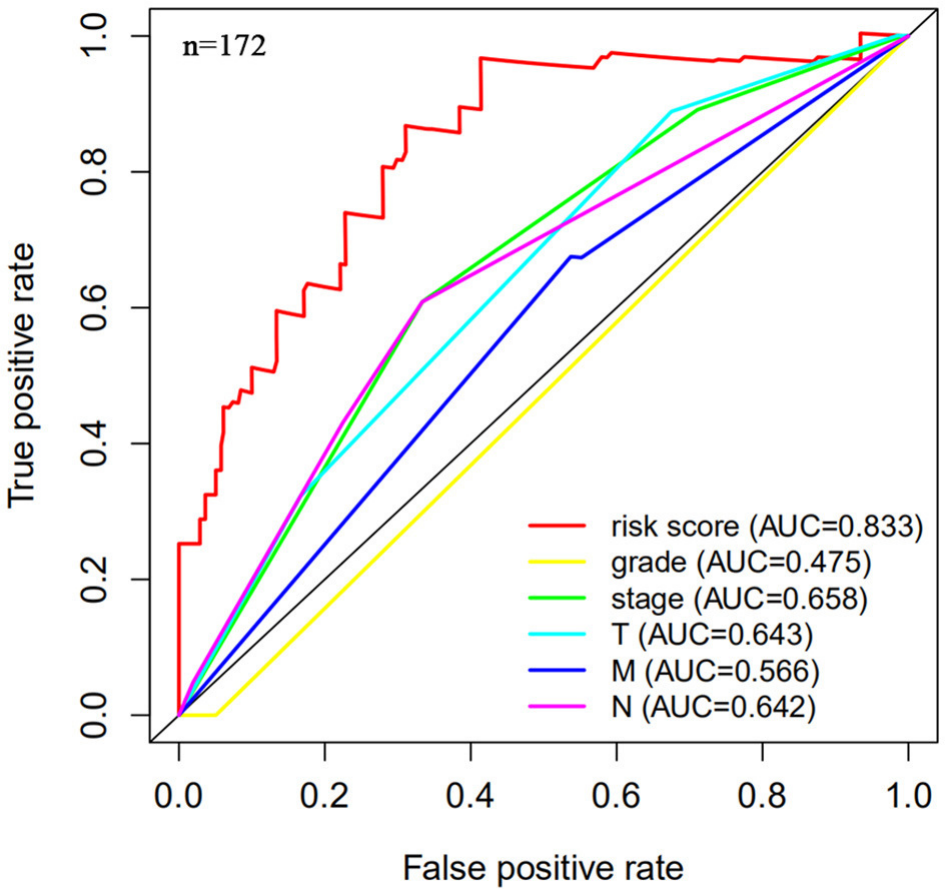
Multi-factor receiver operating curve details. The AUC value of the testing group is significantly more than 0.7, which is greater than the predicted value of clinical data.

Next, this model was validated by using the TCGA testing group. A total of 203 patients with BC were recruited to verify the lncRNA characteristics (Figure 6). We divided the patients with BC into low-risk group and high-risk group based on the median risk score. Consistent with the results obtained from the TCGA data set training group, the survival time was shorter in high-risk patients than in low-risk patients (Figure 7). Testing group data were then screened with complete clinical characteristics (*n* = 172), and the time-related ROC curve was drawn (Figure 8). The AUC value was 0.833, which is >0.7, and indicates that the risk model has great predictive ability and that the predictive ability of the risk model is greater than that of other clinical features. Together, these results show that the established lncRNA signature provides reliable prognostic predictions for patients with BC.

### Gene Set Enrichment Analysis

**Figure 9:**
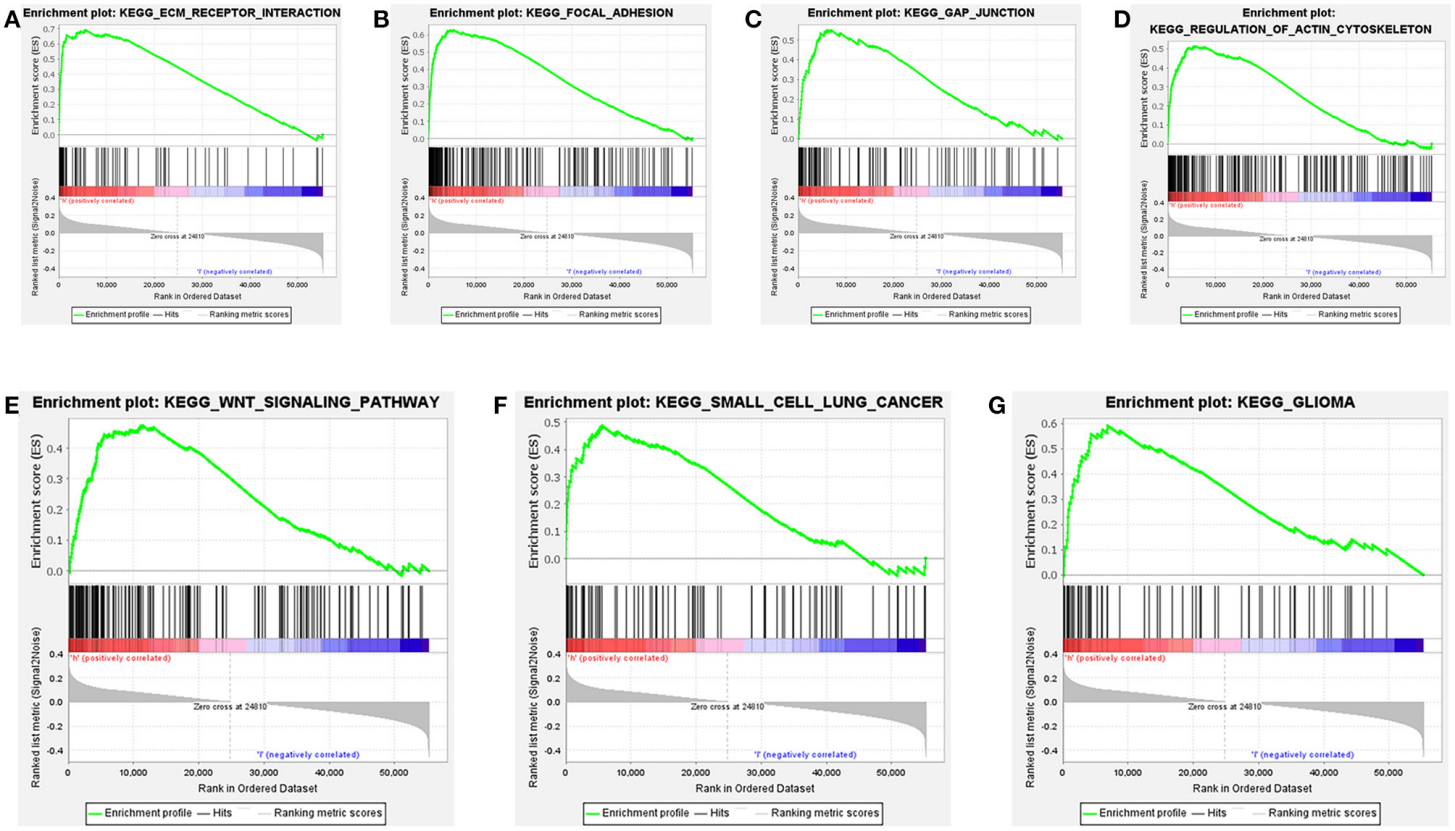
Gene set enrichment analysis (GSEA) based on TCGA data set shows that cancer-related pathways are significantly enriched in highly expressed populations. **(A)** extracellular matrix (ECM) receptor interaction, **(B)** focal adhesion, **(C)** gap junction, **(D)** regulation of actin cytoskeleton, **(E)** wnt signaling pathway, **(F)** small cell lung cancer, **(G)** glioma. The highly expressed population is significantly enriched in ECM receptor interaction, focal adhesion, gap junction, and the regulation of the actin cytoskeleton pathways.

Further functional annotations were made through GSEA. The results showed that differentially expressed genes between the two groups were enriched in tumor-related pathways. A total of 11 gene sets were significantly enriched when the nominal value of *p* < 0.05. We selected seven pathways closely related to tumor metastasis and invasion, including actin cytoskeleton regulation, gap junctions, focal adhesion, and extracellular matrix (ECM) receptor interaction (Table 5). These are important signaling pathways related to tumorigenesis and cancer progression, including in small cell lung cancer, which is highlighted by the relationship between the Wnt signaling pathway and glioma (Figure 9). In conclusion, the identified autophagy-related genes contribute to important cancer pathways. This may provide a new direction for the treatment of BC.

**Table 5:** Gene set enrichment analysis (GSEA) results based on 15 autophagy lncRNA markers.

**Name**	**ES**	**NES**	**NOM *P*-val**	**FDR *q*-val**	**FWER *P*-val**	**RANK AT MAX**	**Leading edge**
KEGG_FOCAL_ADHESION	0.629	2.287	<0.001	0.002	0.003	5254	Tags = 46%, list = 10%, signal = 50%
KEGG_GLIOMA	0.592	2.201	<0.001	0.006	0.007	6855	Tags = 45%, list = 12%, signal = 51%
KEGG_GAP_JUNCTION	0.552	2.060	0.002	0.012	0.039	6855	Tags = 42%, list = 12%, signal = 48%
KEGG_ECM_RECEPTOR_INTERACTION	0.691	2.112	0.002	0.012	0.023	5559	Tags = 56%, list = 10%, signal = 62%
KEGG_REGULATION_OF_ACTIN_CYTOSKELETON	0.515	2.078	<0.001	0.012	0.03	5731	Tags = 38%, list = 10%, signal = 42%
KEGG_WNT_SIGNALING_PATHWAY	0.474	1.927	<0.001	0.025	0.111	11144	Tags = 48%, list = 20%, signal = 60%
KEGG_SMALL_CELL_LUNG_CANCER	0.488	1.864	0.004	0.041	0.181	5555	Tags = 35%, list = 10%, signal = 38%

*ES, enrichment score; FDR, false discovery rate; FWER, familywise-error rate; NES, normalized enrichment score; NOM p-value, nominal p-value*.

## Discussion

Bladder cancer is defined as malignant tumors that occur on the bladder mucosa. BC is the most common malignant tumor of the urinary system (27). There are currently some BC prognostic indicators, including tumor grade, UICC stage, and pTNM stage (28). However, additional prognostic factors are required to more accurately predict and analyze the survival rate in patients with BC. At present, a large number of lncRNAs have been analyzed in the diagnosis and prognosis of various cancers.

Long non-coding RNA Erbb4-IR is downregulated in prostate cancer and can predict the prognosis (29). Overexpression of lncRNA ROR1AS1 predicts a poor prognosis and promotes cervical cancer metastasis by activating the Wnt/β-catenin/EMT signaling cascade (30). lncRNA-D16366 is a potential biomarker for the diagnosis and prognosis of hepatocellular carcinoma (31). lncRNA PVT1 can predict a prognosis in patients with prostate cancer and regulate the tumor growth (32). lncRNA FENDRR and FOXF1 are also prognostic factors for the survival in patients with lung adenocarcinoma (33). In addition, many reports confirm a link between lncRNA and autophagy in cancer.

Reduced expression of lncRNA PRRT3-AS1 can inhibit the prostate cancer cell proliferation and promote apoptosis and autophagy (34). In acute myeloid leukemia, lncRNA UCA1 promotes autophagy by targeting miR-96-5p (35). lncRNA MALAT1 activates autophagy and promotes the cell proliferation by downregulating the miRNA-204 expression in gastric cancer (36). However, to-date, there has been no systematic and comprehensive analysis of autophagy-related lncRNA in BC. This study is the first to use bioinformatics to analyze the value of autophagy-related lncRNA in the diagnosis and prognosis of BC. These results are expected to provide new ideas and research directions for the diagnosis and treatment of BC.

In this study, we collected TCGA data sets and divided them into training group and testing group. In the training group, autophagy-related lncRNAs were used to investigate the prognosis of BC in patients. We identified autophagy-related lncRNAs by constructing a lncRNA autophagy gene co-expression network. We identified a signature of 15 autophagy-related lncRNAs and divided the patients with BC into low-risk group and a high-risk group based on the median risk score. The 5-year survival rate of patients in the high-risk group was shorter than that of patients in the low-risk group. Using univariate and multivariate Cox regression analyses, we showed that the risk score is an independent BC prognostic prediction factor. Subsequently, a multi-factor ROC curve was made, and its AUC was 0.865, which was greater than the AUC of each clinical feature. Together, these results show that the lncRNA signature can better predict a prognosis in patients with BC than can other clinical features. Then, the model was used to predict a prognosis in the testing group, and satisfactory results were obtained, which prove the effectiveness of the model.

Among the 15 autophagy-related lncRNAs, 5 were unfavorable diagnostic factors (AC037198.1, AC084357.2, MAN1B1-DT, AC024060.1, and AC005229.4) and 10 were favorable diagnostic factors for BC (AC026369.3, USP30-as1, AC007991.2, AC104785.1, AC010503.4, AC010331.1, AF131215.6, THUMPD3-AS1, U62317.4, and AL662844.4). The results of GSEA revealed that differentially expressed lncRNAs in high-risk people are enriched in the Wnt signaling, small cell lung cancer, and glioma pathways, gap junctions, focal adhesion, actin cytoskeleton regulation, and ECM receptor interaction.

Previous studies have shown that THUMPD3-AS1 is associated with non-small cell lung cancer and regulates self-renewal through miR-543 and ONECUT2 (37). The Wnt/β-catenin signaling pathway promotes the progression of liver cancer through the activation of SUMO1P3 lncRNA by targeting miR-320a (38). A large number of studies also indicate that lncRNA can directly or indirectly regulate the development of cancer through the regulation of miRNA. Therefore, lncRNA-mRNA co-expression analyses are essential to fully evaluate the function of lncRNAs in cancer. Additionally, the autophagy-related functions of lncRNAs occur through the regulation of mRNA. Therefore, it is important to analyze the specific function of lncRNAs by examining the co-expression of lncRNA-mRNA (39, 40). Ultimately, our results indicate that the 15 autophagy-related lncRNAs identified in this study are potential BC therapeutic targets.

By constructing an autophagy lncRNA co-expression network, we identified a risk model of 15 autophagy-related lncRNAs. Using this model, we were able to separate the group as low risk and high risk based on the median-risk score. This risk score has a prognostic value for patients with BC. In the future, this lncRNA model can be used for risk scoring to predict the prognosis of a patient. However, our research contains several limitations. Bioinformatics methods and public databases were used for this study. Although the stability of the autophagy-related lncRNA signature was successfully validated, the exact molecular mechanisms of these lncRNAs have not been experimentally investigated and their prognostic values have not been experimentally proven. Therefore, further experimental studies using a larger sample size are required to verify these results.

## Data Availability Statement

The data that support the findings of this study are available from the corresponding author upon reasonable request.

## Author Contributions

JW and CG: conceptualization, methodology, validation, formal analysis, investigation, data curation, writing of the original draft, and reviewing and editing. HF, ZX, and YH: validation, formal analysis, investigation, data curation, and visualization. YL: conceptualization, methodology, investigation, data curation, reviewing and editing the manuscript, visualization, supervision, and project administration. All authors: agree to be accountable for the content of the work.

## Conflict of Interest

The authors declare that the research was conducted in the absence of any commercial or financial relationships that could be construed as a potential conflict of interest.
